# Cerebral malaria caused by *Plasmodium vivax* in adult subjects

**DOI:** 10.4103/0972-5229.45084

**Published:** 2008

**Authors:** Suman Sarkar, Prithwis Bhattacharya

**Affiliations:** **From:** Department of Anesthesiology, I.M.S. Banaras Hindu University, Varanasi - 221 105, Uttar Pradesh, India

**Keywords:** Cerebral malaria, meningoencephalitis, *Plasmodium vivax*

## Abstract

Cerebral malaria is a diffuse encephalopathy associated with seizures and status epilepticus which can occur in up to one-third of patients with severe malaria, particularly that caused by *Plasmodium falciparum*.

In this article, we report three cases of *Plasmodium vivax* malaria (all adult male patients) complicated by seizures and symptoms of diffuse meningoencephalitis. Two patients had predominantly meningeal signs, while in the third patient the features were purely of encephalitis All cases were treated with artesunate. Usually, cerebral malaria is caused by *P. falciparum*, and rarely, cerebral malaria is a presenting complication or occurs during the course of *P. vivax* infection.

## Introduction

Cerebral malaria is usually secondary to *P. falciparum* infection. However, there are infrequent reports of cerebral malaria associated with *P. vivax* infection. To our knowledge, only 45 cases of central nervous system *P. vivax* malaria are reported in the scientific literature since 1920, about half of these cases have occurred in children.[1,2]

Here we report three rare cases of cerebral malaria caused by *P. vivax*.

## Case Report

Three male patients, each aged over 18 years, presented with high-grade intermittent fever of more than four days duration. The fever was associated with chills and rigors. All the three patients had presented with altered consciousness There was history of generalized tonic clonic convulsions prior to admission, in each case. Two of them were severely dehydrated. Their capillary blood sugar level was normal at presentation.

In all cases, routine blood counts, liver function tests, and serum electrolytes, serum urea, and serum creatinine were within normal limits. Peripheral blood smear revealed trophozoites of *P. vivax*. Antigen test for *P. vivax* was positive, while that for *P. falciparum* was negative in all cases. Their cerebrospinal fluid and electroencephalogram (EEG) findings were unremarkable. The patients were treated with supportive therapy and intravenous Artesunate in the recommended dose. Repeat blood smears after two days of therapy showed clearance of the parasites. All the three patients were discharged from ICU in a clinically stable condition and were advised to take Primaquine for 14 days. Follow-up evaluation after one month showed no residual neurological deficit.

## Discussion

Organ dysfunction characteristic of *P. falciparum* malaria is unusual in *P. vivax* infections. Any patient infected with *P. viva* who exhibits severe malaria is presumed to be suffering from mixed infection.[2] However, that may not be always true. As evident from the present report, *P. vivax* infection can also present as cerebral malaria. Clinical data provided by Kochar *et al*, indicates that *P. vivax* can cause both sequestration-related and nonsequestration-related complications of severe malaria, all of which are commonly associated with *P. falciparum* infections.[3] The exact pathogenetic mechanism however remains elusive. Sachdev and Mohan[4] studied the clinicolaboratory profile of six patients with *P. vivax* cerebral malaria. The presenting features were of acute febrile encephalopathy, convulsions, and coma. Focal neurological signs were observed in one patient. Ozen *et al*,[1] have recently described a case of cerebral *P. vivax* malaria that presented with status epilepticus. Some experts also suggest that cerebral malaria subjects might have an underlying seizure disorder and those seizures are precipitated by the high fever associated with the disease.
